# The Japanese Breast Cancer Society Clinical Practice Guideline for radiation treatment of breast cancer, 2018 edition

**DOI:** 10.1007/s12282-019-01019-5

**Published:** 2019-10-28

**Authors:** Chikako Yamauchi, Michio Yoshimura, Kenji Sekiguchi, Yasushi Hamamoto, Naomi Nakajima, Naoko Sanuki, Etsuyo Ogo, Masahiko Oguchi, Shigehira Saji, Hiroji Iwata

**Affiliations:** 1 Department of Radiation Oncology, Shiga General Hospital, 5-4-30 Moriyama, Moriyama-shi, Shiga 524-8524 Japan; 2 Department of Radiation Oncology and Image-applied Therapy, Kyoto University Graduate School of Medicine, Kyoto, Japan; 3 Department of Radiation Oncology, Sonoda-kai Radiation Oncology Clinic, Tokyo, Japan; 4 Department of Radiology, Ehime University Hospital, Toon, Japan; 5 Radiation Oncology Department, The Cancer Institute Hospital, Japanese Foundation for Cancer Research, Tokyo, Japan; 6 Radiation Therapy Department, Mie Prefectural General Hospital, Yokkaichi, Japan; 7 Department of Radiology, Kurume University School of Medicine, Kurume, Japan; 8 Department of Medical Oncology, Fukushima Medical University, Fukushima, Japan; 9 Department of Breast Oncology, Aichi Cancer Center Hospital, Nagoya, Japan

## Abstract

**Purpose:**

The Japanese Breast Cancer Society (JBCS) Clinical Practice Guideline was revised in 2018. This article describes the revise points in the section on radiation therapy (RT).

**Methods and materials:**

The JBCS formed task force to update the JBCS Clinical Practice Guideline 2015 edition. Background questions (BQs) deal with standard treatments of breast cancer in clinical practice. Clinical questions (CQs) highlight the important treatments in which controversy remains. The task force for RT section addressed the 10 BQs, the 10 CQs, and the 4 Future reseach questions (FQs). For each CQ, systematic literature reviews and meta-analyses were conducted, and recommendations, strength of recommendation and strength of evidence were determined according to the protocol in Morizane et al. (Minds Handbook for Clinical Practice Guideline Development, 2014).

**Results:**

The recommendations, the strength of recommendation and the strength of evidence were determined based on the systematic literature reviews and the meta-analyses for each CQ.

**Conclusion:**

The JBCS updated the Clinical Practice Guideline. RT represents a significant portion of the breast cancer treatment, and these recommendations regarding RT will be useful in individualized, shared decision making between physicians and patients.

## Introduction

The Japanese Breast Cancer Society (JBCS) Clinical Practice Guideline was revised in 2018. In the 2018 edition, the concepts and the methods used for the guideline were significantly changed according to the protocol in Minds Handbook for Clinical Practice Guideline Development (2014) [1]. This article describes the revise points and adds the short explanation for each CQ in the section on radiation therapy (RT).

**BQ1**. Is whole breast irradiation (WBI) following breast-conserving surgery (BCS) recommended for patients with stage I–II breast cancer?

**Statement**

Whole breast irradiation is the standard treatment.

**BQ2**. Is RT recommended for patients with ductal carcinoma in situ (DCIS) after BCS?

**Statement**

Whole breast irradiation is the standard treatment.

**BQ3**. Is RT following BCS recommended for patients with a pathologic complete response (pCR) after neoadjuvant chemotherapy?

**Statement**

Whole breast irradiation is the standard treatment.

**CQ1**. In WBI, is hypofractionated RT recommended as an equivalent treatment than conventional fractionation?

**Recommendation**

For patients aged > 50 years, with pT1-2N0, and without chemotherapy, hypofractionated WBI is strongly recommended [strength of recommendation (SoR): 1, strength of evidence (SoE): moderate].

For patients other than those described above, hypofractionation is weakly recommended because the data are still not sufficient [SoR: 2, SoE: weak].

With regard to the dose and fractionation for WBI, a total dose of 45–50.4 Gy in 25–28 fractions over a period of 4.5–5.5 weeks has been conventionally used. From the results of the randomized control trials (RCTs) performed in Canada [2] and the United Kingdom [3], hypofractionated WBI for about 3 weeks has been applied to many patients with conserved breast instead of the conventional fractionation. In 2011, the American Society for Radiation Oncology (ASTRO) developed a guideline demonstrating that the hypofractionation method is equivalent to the conventional method used in patients aged > 50 years with pT1-2N0 tumors, without systemic chemotherapy, and with no more than ± 7% dose homogeneity in the central axis plane, and that it is not contraindication in other patients [4]. In this revision, we conducted a systematic review of the RCTs of hypofractionation. Compared with conventional fractionation, the risk ratios (RRs) of hypofractionation were not significantly different in regional lymph node recurrence (RR 1.24, 95% CI 0.56–2.77), local recurrence (RR 0.93, 95% CI 0.77–1.12), distant recurrence (RR 1.1, 95% CI 0.72–1.68), and overall survival (RR 0.92, 95% CI 0.82–1.04). With regard to late adverse events, RRs of radiation pneumonitis (RR 0.98, 95% CI 0.14–6.96), breast fibrosis (RR 0.93, 95% CI 0.83–1.05), rib fracture (RR 0.87, 95% CI 0.25–3.10), cosmesis (RR 1.11, 95% CI 0.99–1.24) and ischemic heart disease (RR 0.71, 95% CI 0.28–1.79) were not significantly different. Based on these findings, we presumed that the degree of recommendation is stronger in this edition than that of the 2015 edition, although further long-term follow-up results of patients with heart disease (one of the serious late effects) who received hypofractionation are warranted. Due to the differences in physical constitution among races, the degree of adverse events caused by hypofractionated WBI may differ and, thus, the Japanese Clinical Oncology Group (JCOG) 0906 was conducted as a single arm trial of over 300 patients. According to the results of a primary analysis with a relatively short follow-up period of 3 years, hypofractionated WBI can be safely performed in Japanese women with acceptable acute and late effects on normal tissues [5]. In 2018, ASTRO updated the guideline for WBI, and the hypofractionated WBI is recommended for all patients with invasive breast cancer with or without inclusion of the low axilla regardless of age, stage, and chemotherapy [6]. On the contrary, we retained the three criteria, although the possible removal of these criteria was discussed in the task force members. This is due to the fact that most of the patients in the Canadian study with the longest follow-up periods met the three criteria, and no previous study has provided sufficient evidence regarding the efficacy of this method since 2011. In summary, the effects and the adverse events of conventional fractionation and the hypofractionation seem to be equivalent, although further long-term observation on the cardiac events is necessary. In addition, the hypofractionated WBI is not consuming time and cost effective. If there is no difference in safety and effectiveness, it is highly convenient to complete the treatment in a shorter period. Therefore, after considering patient selection, dose homogeneity, and dose to normal tissues such as the heart, hypofractionated WBI is recommended.

**CQ2**. Is boost irradiation for the tumor bed recommended following WBI in the patients with negative surgical margin after BCS?

**Recommendation**

Boost irradiation to the tumor bed is weakly recommended for patients with pathologically negative margins who underwent BCS for invasive breast cancer [SoR: 2, SoE: moderate].

In patients who underwent pathologically complete excision for invasive disease, an RCT conducted by the European Organization for Research and Treatment of Cancer (EORTC) showed that delivering a 16 Gy boost to the tumor bed reduced the rate of ipsilateral breast tumor recurrence from 16.4 to 12.0% [7]. There was a significant decrease in the local recurrence rate in the boost group: 40 years or younger, 41–50 years, 51–60 years, 61 years or older; the absolute risk reduction was particularly significant in patients aged 40 years or younger. On the contrary, the surgical margin is considered negative even if the intraductal component is present at the inked margin of the surgical specimen. Compared with Japan, the impact of boost irradiation might be stronger than in Japan because the definition of negative margin is different from Japan.

Although the cumulative incidence of severe fibrosis at 20 years was 5.2% in the no boost group versus 1.8% in the boost group, the absolute risk rate was not high and there was no significant difference in the frequency of severe fibrosis between both groups in the younger age group (40 years or younger). The risk of severe fibrosis is even lower in patients who received a 10 Gy boost irradiation, which is the standard boost dose in Japan. In addition to the above points, after considering the prolonged treatment time and the costs, a boost irradiation to the tumor bed is weakly recommended in patients with a negative surgical margin.

**CQ3**. Is accelerated partial breast irradiation (APBI) recommended after BCS?

**Recommendation**

Long-term adequate evidence is not sufficient; hence, APBI is weakly recommended not to perform [SoR: 3, SoE: moderate].

According to the 2016 Cochrane systematic review of APBI [8], the rate of local recurrence after APBI was higher than that after WBI, although the overall survival, breast cancer death, and distant recurrence rates were not significantly different compared to WBI. However, studies on intraoperative irradiation reported higher rate of local recurrence, which accounted for about 70% of the total cases, and no significant difference was found when the results of studies on brachytherapy and external beam irradiation were analyzed. Based on the results of our meta-analysis with the addition of a new paper, the risk ratio (RR) was 0.99 (95% CI 0.55–1.78, *p* = 0.97) for cosmesis, and 0.60 (95% CI 0.34–1.07, *p* = 0.08) for late skin adverse effect, but a statistically significant difference was not found. The results differ depending on the methods of APBI, and the observation period was not sufficient. Furthermore, only a few studies in Japan have evaluated the effects of APBI. Hence, it is necessary to consider the differences in physique and breast size when employing this method in the Western population. In addition, APBI is not commonly performed in Japanese facilities, and it remains unclear whether APBI is truly non-inferior compared with WBI about in terms of long-term treatment results and adverse events. Therefore, APBI is weakly recommended not to do.

**BQ4**. Is regional node (supraclavicular region) irradiation recommended for patients with four or more positive axillary nodes after BCS?

**Statement**

RT to the ipsilateral supraclavicular node is the standard treatment.

**CQ4**. Is regional node (supraclavicular region) irradiation recommended for patients with 1–3 positive axillary nodes after BCS?

**Recommendation**

Although RT to the ipsilateral supraclavicular node is not routinely recommended, it is weakly recommended for patients with high-risk factors [SoR: 2, SoE: weak].

Two RCTs (MA.20 Trial [9] and EORTC 22922 [10]) included patients who underwent BCS and postoperative RT and examined the usefulness of the regional lymph node (supraclavicular) irradiation in addition to WBI. Both trials partially included those patients with four or more lymph node metastases. All the 1832 patients in MA.20 and 76% of the 4004 patients in EORTC 22922 received BCS. In both trials, although the 10-year distant metastasis-free survival rate significantly improved, no significant difference was observed in the 10-year overall survival rate. According to the results of the meta-analysis of these two trials, although the distant recurrence rate was significantly reduced (RR 0.81, 95% CI 0.72–0.90, *p* = 0.0002), the reduction of regional lymph node recurrence rate and the improvement of overall survival rate were not statistically significant. The risk of adverse events was evaluated by adding observational studies to RCT. Lymphedema was significantly increased (RR 2.6, 95% CI 1.64–4.10, *p* < 0.0001), but there was no significant difference in secondary malignancy or cardiotoxicity. The patients’ preference after considering the benefits and harms varied individually. Based on these findings, RT to ipsilateral supraclavicular node is not routinely recommended and it is weakly recommended in the patients with high-risk factors. The possible risk factors are lymphovascular invasion, extracapsular invasion, a large number of lymph node metastases (2 than 1 and 3 than 2), high nuclear grade, negative hormone receptor, and larger tumor size, although there is no sufficient evidence. In addition, the overlapping of these risk factors may increase the significance of regional lymph node irradiation.

**BQ5**. Is postmastectomy radiation therapy (PMRT) recommended for patients with 4 or more positive axillary lymph nodes after mastectomy?

**Statement**

Postmastectomy radiation therapy is the standard treatment.

**CQ5**. Is PMRT recommended for patients with 1–3 positive axillary lymph nodes after mastectomy?

**Recommendation**

Postmastectomy radiation therapy is recommended [SoR: 1–2, SoE: moderate, Consensus: not agreed].

Of 22 RCTs on PMRT, the EBCTCG meta-analysis analyzed 1314 patients with 1–3 positive axillary node metastases [11]. The 10-year locoregional recurrence rate of patients who underwent PMRT decreased to 3.8% compared with that of the no PMRT group (20.3%) (RR 0.24, 95% CI 0.17–0.34, 2*p* < 0.00001). The 10-year overall recurrence rate decreased from 45.7 to 34.2% after performing PMRT (RR 0.68, 95% CI 0.57–0.82, 2*p* = 0.00006), and the 20-year breast cancer death rate also decreased significantly from 50.2 to 42.3% (RR 0.80, 95% CI 0.67–0.95, 2*p* = 0.01). Although the 20-year all-cause mortality rate was 53.5% with PMRT and 56.5% without PMRT, no statistically significant difference was observed (RR 0.89, 95% CI 0.77–1.04). Late adverse events were assessed by conducting a systematic review of patients with heart disease and secondary malignancy [12]. Although long-term follow-up has been carried out, it is necessary to keep in mind that old irradiation techniques were used in the studies included in the systematic review. Based on the review, the mortality rate from heart diseases without breast cancer recurrence increased (RR 1.3, 95% CI 1.15–1.46, *p* < 0.001). With regard to secondary cancer without prior breast cancer recurrence, the incidence of contralateral breast cancer was 0.45% in the RT group and 0.37% in the no RT group (RR 1.20, *p* < 0.001), while the incidence of lung cancer after 10 years was 0.05% in the RT group and 0.02% in no RT group (RR 2.10, *p* < 0.001). The incidence of all secondary cancers except breast cancer was 0.50% in the RT group and 0.42% in the non-RT group (RR 1.23, *p* < 0.001). For arm lymphedema, we conducted a meta-analysis of two cohort studies, the EORTC 22922/10925 study that reported a 3-year adverse event [13] and a prospective cohort study from the Massachusetts General Hospital [14]. Result showed that the incidence of lymphedema increased (RR 2.71, *p* = 0.30) in patients who underwent RT, including those who received regional node and occurred in 10–20% of the total cohort, although it was not statistically significant. Lung and skin toxicities were reported in the EORTC 22922/10925 study mentioned previously. According to the results, pulmonary fibrosis, radiation pneumonitis, all lung toxicity, and all skin toxicity (dermatitis, dermal fibrosis, pigmentation, telangiectasia, etc.) occurred within 3 years in 2.8%, 0.7%, 4.3%, and 13.6% of patients, respectively, who were irradiated at the internal mammary and medial supraclavicular nodes aside from the chest wall or breast.

Postmastectomy radiation therapy increased the rate of mortality from heart disease and the incidence of secondary cancer and lymphedema, although the magnitude of the differences was small. For patients who underwent PMRT, hospital visits could be time consuming and the treatment itself could be costly, which caused a significant burden to these patients. Adverse events depend on age, the presence of comorbidities, tumor localization, obesity, etc., and the patient’s own assessment of adverse events varies depending on individual values. The cost of performing PMRT is high; if the disease recurs, the cost of treatment is even higher, which can have negative effects on the patients’ physical and mental aspect Therefore, the patients’ preference to receive PMRT will likely vary. The evaluated studies were conducted before new drugs, such as aromatase inhibitors, trastuzumab and taxanes, were used. Therefore, the significance of PMRT may be diminished at the time when these drugs were standardly administered. On the contrary, there are also reductions in the adverse events due to the advances in RT technology. Foreign guidelines strongly recommend PMRT, but there may be subgroups of patients who can be omitted PMRT. With regard to the strength of the recommendation, the opinions of the panel were divided into two categories by voting as follows: whether it was strongly or weakly recommended. By voting, the strength of the recommendation could not be determined.

**BQ6**. Is chest wall irradiation recommended for patients who underwent PMRT?

**Statement**

Chest wall irradiation is the standard treatment.

**BQ7**. Is supraclavicular nodal irradiation recommended in patients who underwent PMRT?

**Statement**

Supraclavicular nodal irradiation is the standard treatment.

**CQ6**. Is it recommended to include the internal mammary nodes (IMNs) in patients with positive axillary lymph node metastases and in those who underwent regional node irradiation (RNI) after BCS or mastectomy?

**Recommendation**

It is weakly recommended to include IMNs [SoR: 2, SoE: weak].

Although the recurrence rate of IMNs is low even if RNI is omitted, IMNs were included in the RCTs showing that RNI can improve patients’ survival [15, 16]. An RCT conducted in France reported the significance of performing IMN irradiation as an additional treatment [17]. The trial included 1334 patients with positive axillary LN metastasis or primary lesion in the inner/medial area. The median follow-up period was 11.3 years; there was no significant difference in the 10-year survival rate. However, it should be considered that about 85% of the patients had T1–2 tumor and about 25% had no LN metastasis. Moreover, an old-fashioned two-dimensional treatment plan was used in this trial. A Danish prospective cohort study (DBCG-IMN) included 3089 patients [18]. In this study, the results of node-positive patients with right-sided cancers who received IMN irradiation in addition to breast or chest wall and SC node irradiation were compared with the results of patients with left-sided cancers who did not undergo IMN irradiation. Approximately, 35% of the patients received BCS, while about 65% underwent mastectomy. At a median follow-up of 8.9 years, the overall survival rate improved significantly from 75.9% in the irradiated group to 72.2% in the non-irradiated group (hazard ratio [HR] 0.82, *p* = 0.005). Breast cancer mortality rate also decreased significantly (20.9% vs 23.4%, HR 0.85, *p* = 0.03), and distant metastasis rates also tended to decrease (27.4% vs 29.7%, HR 0.89, *p* = 0.07). Especially in patients with four or more axillary LN metastases, IMN irradiation improved the overall survival rate. In this study, three-dimensional treatment planning using computed tomography was performed in many cases.

Two RCTs (MA.20 Trial [9] and EORTC 22922 Trial [10]) evaluated the significance of performing RNI in addition to IMN, although they did not directly verify the significance of IMN irradiation. RNI significantly reduced locoregional recurrence; however, it did not improve overall survival in both trials. Results of the meta-analysis of these two trials indicate that RNI reduced the locoregional recurrence rate (RR 0.81, *p* = 0.02), distant metastasis rate (RR 0.80, *p* = 0.0002), and overall mortality rate (RR 0.90, *p* = 0.05). However, the two trials included patients who had SC and IMN irradiation and those who did not undergo RNI; hence, the effects of treating these two sites could not be separately evaluated.

With regard to adverse events, the number of grade 3/4 late adverse events has not increased in the French trial. Moreover, the observation period was not sufficient to note for cardiac-related adverse events, and data for further long-term observation are required. However, there was no significant difference in the incidence of cardiac events between the control and RNI arm. On the contrary, RNI including IMN increases radiation pneumonitis. For late pulmonary adverse events, although the methods of evaluation varied, pulmonary fibrosis (any grade) occurs in 4% of the patients who received RNI in the EORTC 22922 trial [10]. For those patients who underwent RNI, the addition of IMN had no significant impact on the hospital and treatment costs. IMN irradiation is not recommended in all patients who underwent RNI; however, it should be performed in high-risk patients. There is not enough evidence to identify high-risk patients such as clinically or pathologically positive IMN metastasis, four or more positive axillary lymph node metastases, or one to three positive axillary LN metastases from medial/central primary tumors.

**CQ7**. Is PMRT recommended for patients who responded to neoadjuvant systemic therapy (NAST)?

**Recommendation**

Postmastectomy radiation therapy is weakly recommended even for the patients who responded to NAST based on the indication of the pretreatment stage [SoR: 2, SoE: very weak].

Although no prospective RCT has reported the use of PMRT after NAST, the evidence is not sufficient to support the use of this method in patients who responded well to NAST; the results of several retrospective studies have been reported. A report of the integrated analysis of the NSABP B-18 and B-27 studies showed that the clinical stage and the therapeutic effect of the primary lesion and LN (pCR or ypN0) are significant predictors of locoregional recurrence in patients who underwent mastectomy after NAST [19]. In a retrospective analysis of the data from six prospective clinical trials conducted at the M.D. Anderson Cancer Center, 11% of 542 patients with PMRT and 22% of 134 patients without PMRT had locoregional recurrence up to 10 years (*p* = 0.0001), although the 10-year overall survival rate was not significantly different [20]. In a retrospective study from the same facility, 106 patients who underwent mastectomy and achieved pCR with NAST were analyzed. In the patients with clinical stage I–II, both groups of patients with or without PMRT had no locoregional recurrence up to 10 years, but the stage III patients without PMRT had significantly higher locoregional recurrence rates than those with RT (7.3 ± 3.5% vs 33.3% ± 15.7%, *p* = 0.040). On the contrary, a study of the patients with stage II–III breast cancer who achieved ypN0 with NAST showed different results. The study included 151 patients with ypN0, of whom 105 underwent PMRT. In multivariate analysis, PMRT did not contribute to disease-free survival rate, locoregional recurrence-free survival rate and overall survival rate [26]. Although these studies did not provide a detailed description of the adverse events, it is necessary to consider possible occurrence of similar adverse events and the costs of PMRT without preoperative chemotherapy (see CQ5). With regard to the patients’ preference, they may wish to omit PMRT if NAST is successful because the length of treatment and the treatment costs may increase. The results of previous studies are biased in terms of the background and the methods of treatment; hence, the strength of evidence is considered “very weak”. At present, it is weakly recommended to determine the indication of PMRT according to the stage prior to NAST even if NAST is effective.

**CQ8**. Is PMRT recommended for the patients who underwent mastectomy and breast reconstruction?

**Recommendation**

**CQ8a**. For the patients with autologous reconstructed breast, PMRT is strongly recommended [SoR: 2, SoE: weak].

Most studies on PMRT after reconstruction using autologous tissue are retrospective. We conducted a meta-analysis of six studies for adverse events related to reconstructed breasts using autologous tissue (291 patients with PMRT and 1003 patients without PMRT). In the irradiated group, odds ratio of the adverse events on reconstructed breasts was 1.11 compared with that of the non-irradiated group. On the contrary, PMRT has certain adverse effects (see CQ5); hospital visits are time consuming and the treatment is expensive. At present, the benefits of PMRT are likely to outweigh the harm, although there is not enough information in the safety of PMRT for reconstructed breasts with autologous tissue transplantation.

**CQ8b**. For patients with implant reconstruction, PMRT is weekly recommended [SoR: 2, SoE: weak].

Most studies reporting the use of PMRT after reconstruction using implants are retrospective, and the adverse events reported, such as capsular contractures, implant deviations, pain, and infections, are inconsistent. We conducted a meta-analysis of two studies to determine the adverse events related to reconstructed breasts using implants (428 patients with PMRT and 1912 patients without PMRT), and the severe adverse events resulted in an odds ratio of 9.32 (95% CI 1.57–55.36, *p* = 0.01) in the irradiated group compared with the non-irradiated group. Breast reconstruction is performed even in patients with high risk of recurrence; many patients prefer implant-based reconstruction in which the surgical wound is limited to the breast. Because PMRT significantly increases the incidence of adverse events, the benefit should be valued over the harm in patients indicated for PMRT. RT can be safely performed in patients who underwent implant-based breast reconstruction by careful judgment and management; thus, PMRT of the implant reconstructed breasts is weakly recommended, as there is insufficient evidence supporting its safety at this time.

**CQ8c**. For patients with a temporary tissue expander, PMRT is strongly recommended not to do [SoR: 4, SoE: weak].

Several studies reported that the incidence of complications increases when PMRT is performed, while the expander is inserted, than when the implant is irradiated. The perturbation in dose distribution around the metallic port for the saline injection of the expander may be compromised. We conducted a meta-analysis of three studies (161 patients with RT and 475 patients without RT) to determine the adverse events caused by irradiation to the expander. The odds ratio of the irradiated group is 23.41 (95% CI 2.83–193.43, *p* = 0.0007) compared to non-irradiated group. Furthermore, we performed a meta-analysis of five studies evaluating the timing of RT for the patients with implant-based breast reconstruction; the studies included 306 patients who were irradiated during the expander insertion and 206 patients irradiated after the implant replacement. The patients who were irradiated during the expander insertion had a significantly higher reconstruction failure rate with an odds ratio of 3.17 than the patients who were irradiated after the implant replacement. Based on the above results, irradiation to the expander is thought to increase adverse events, and the benefits of radiation therapy may also be reduced due to the effects of port metal. Therefore, PMRT to the expander is not recommended and if necessary, PMRT following the replacement to the implant is recommended.

**BQ8**. What is the appropriate timing for RT after breast surgery?

**Statement**

For the patients who do not undergo postoperative chemotherapy, RT should be initiated no more than 20 weeks after surgery.

For the patients who undergo postoperative chemotherapy, chemotherapy should be performed prior to RT.

Concurrent RT and chemotherapy are not fundamentally recommended.

Concurrent RT and endocrine therapy may be considered when deemed necessary.

Concurrent RT and anti-HER2 therapy should be performed with careful attention to the occurrence of cardiovascular adverse events if the radiation fields include the heart.

**BQ9**. Is RT recommended for painful bone metastasis of breast cancer?

**Statement**

RT is the standard treatment for painful bone metastases.

**CQ9**. Is a single 8 Gy administration of RT recommended for painful bone metastasis of breast cancer?

**Recommendation**

A single 8 Gy administration of RT is weakly recommended [SoR: 2, SoE: moderate].

As palliative RT for painful bone metastases, 30 Gy in 10 fractions has been frequently used. However, the effectiveness and safety of single fraction 8 Gy remain unclear. Based on the results of previous RCTs and systematic reviews, the administration of a single fraction of 8 Gy is recommended in Western guidelines [21, 22]. We conducted a meta-analysis of the relatively large RCT (more than 100 cases in both single and multifraction groups) that evaluated the usefulness of single fraction RT. The pain relief rates were similar in both arms (RR 1.00, 95% CI 0.95–1.05, *p* = 0.97). With regard to the incidence of spinal cord compression, pathological fractures, and re-irradiation, the RRs were 1.42 (95% CI 0.88–2.29, *p* = 0.15), 1.16 (95% CI 0.63–2.13, *p* = 0.64), and 2.37 (95% CI 1.65–3.40, *p* < 0.00001), respectively. Although there were no statistically significant differences in acute adverse events of Grade 2 or higher, the RR was 0.73 (95% CI 0.53–1.00, *p* = 0.05), and the single fraction tended to cause fewer acute adverse events. Single fraction RT is recommended as it is convenient and economical for patients, and pain relief effect is equivalent to that of multifraction RT. However, the possibility of re-irradiation and the risk of spinal cord compression and fractures should be considered in the long-term prognosis of patients.

**BQ10**. Is RT recommended for brain metastasis of breast cancer?

**Statement**

RT is the standard treatment.

**CQ10**. Is stereotactic radiosurgery (SRS) without whole-brain irradiation (WBI) as an initial treatment recommended for patients with brain metastasis of breast cancer, when good prognosis can be expected, when the maximum diameter of all brain metastasis lesions are less than 3 cm, and when the number of brain metastases is 1–4?

**Recommendation**

It is weakly recommended to perform SRS as an initial treatment and to omit WBI until the brain metastasis progresses, which is beyond the indication for SRS [SoR: 2, SoE: moderate].

We considered whether SRS alone or addition of WBI to SRS as an initial treatment is appropriate for patients with good prognosis, the maximum diameter of all brain metastasis lesions was less than 3 cm and with 1–4 brain metastases, (including brain metastasis of solid cancers other than breast cancer), based on the RCTs [23–25]. The meta-analysis of these trials revealed that the RR of SRS alone was 1.01 (95% CI 0.93–1.10, *p* = 0.94) compared with SRS + WBI and was not significantly different. With regard to the intracranial progression rate, the RR of SRS alone was 2.35 (95% CI 1.78–3.11, *p* < 0.00001), and the intracranial recurrence rate decreased significantly after adding WBI. The higher brain dysfunction rate was assessed using Hopkins Verbal Learning Test-Revised (HVLT-R) in two RCTs, and the RR of the SRS alone group was 0.63 (95% CI 0.51–0.78, *p* = 0.11). The initial treatment of SRS alone resulted in a significantly high rate of intracranial progression. However, it prevents the occurrence of hair loss and higher brain dysfunction without compromising survival. It is weakly recommended to treat 1–4 brain metastases using SRS alone in patients with good prognosis and to avoid using WBI until the brain metastasis progresses which is beyond the indications for SRS.
