# Correction to: The Japanese Breast Cancer Society Clinical Practice Guideline for radiation treatment of breast cancer, 2018 edition

**DOI:** 10.1007/s12282-021-01255-8

**Published:** 2021-05-19

**Authors:** Chikako Yamauchi, Michio Yoshimura, Kenji Sekiguchi, Yasushi Hamamoto, Naomi Nakajima, Naoko Sanuki, Etsuyo Ogo, Masahiko Oguchi, Shigehira Saji, Hiroji Iwata

**Affiliations:** 1 Department of Radiation Oncology, Shiga General Hospital, 5-4-30 Moriyama, Moriyama-shi, Shiga 524-8524 Japan; 2 Department of Radiation Oncology and Image-applied Therapy, Kyoto University Graduate School of Medicine, Kyoto, Japan; 3 Department of Radiation Oncology, Sonoda-kai Radiation Oncology Clinic, Tokyo, Japan; 4 Department of Radiology, Ehime University Hospital, Toon, Japan; 5 Radiation Oncology Department, The Cancer Institute Hospital, Japanese Foundation for Cancer Research, Tokyo, Japan; 6 Radiation Therapy Department, Mie Prefectural General Hospital, Yokkaichi, Japan; 7 Department of Radiology, Kurume University School of Medicine, Kurume, Japan; 8 Department of Medical Oncology, Fukushima Medical University, Fukushima, Japan; 9 Department of Breast Oncology, Aichi Cancer Center Hospital, Nagoya, Japan

## Correction to: Breast Cancer (2020) 27:9–16 10.1007/s12282-019-01019-5

The article “The Japanese Breast Cancer Society Clinical Practice Guideline for radiation treatment of breast cancer, 2018 edition”, written by Chikako Yamauchi, Michio Yoshimura, Kenji Sekiguchi, Yasushi Hamamoto, Naomi Nakajima, Naoko Sanuki, Etsuyo Ogo, Masahiko Oguchi, Shigehira Saji, Hiroji Iwata, was originally published electronically on the publisher’s internet portal on 28 October 2019 without open access. After publication in volume 27, issue 1, page 9–16, the author decided to opt for Open Choice and to make the article an Open Access publication. Therefore, the copyright of the article has been changed to © The Author(s) and the article is forthwith distributed under the terms of the Creative Commons Attribution 4.0 International License, which permits use, sharing, adaptation, distribution and reproduction in any medium or format, as long as you give appropriate credit to the original author(s) and the source, provide a link to the Creative Commons licence, and indicate if changes were made.

The images or other third party material in this article are included in the article’s Creative Commons licence, unless indicated otherwise in a credit line to the material. If material is not included in the article’s Creative Commons licence and your intended use is not permitted by statutory regulation or exceeds the permitted use, you will need to obtain permission directly from the copyright holder.

To view a copy of this licence, visit http://creativecommons.org/licenses/by/4.0/.

The original article has been updated.

